# The patient-level effect of the cost of Cancer care – financial burden in German Cancer patients

**DOI:** 10.1186/s12885-020-07028-4

**Published:** 2020-06-05

**Authors:** Katja Mehlis, Julian Witte, Bastian Surmann, Matthias Kudlich, Leonidas Apostolidis, Jürgen Walther, Dirk Jäger, Wolfgang Greiner, Eva C. Winkler

**Affiliations:** 1 National Center for Tumor Diseases (NCT), Department of Medical Oncology, Program for Ethics and Patient-Oriented Care, Heidelberg University Hospital, Im Neuenheimer Feld 460, 69120 Heidelberg, Germany; 2 Department for Health Economics and Health Care Management, Bielefeld University, School of Public Health, PO box 10 01 31, 33501 Bielefeld, Germany

**Keywords:** Financial burden, Financial toxicity, Out-of-pocket costs, Income loss, Patient-reported outcomes, Quality of life

## Abstract

**Background:**

Financial toxicity of cancer has so far been discussed primarily in the US health care system and is associated with higher morbidity and mortality. In European health care systems, the socio-economic impact of cancer is poorly understood. This study investigates the financial burden and patient-reported outcomes of neuroendocrine (NET) or colorectal (CRC) cancer patients at a German Comprehensive Cancer Center.

**Methods:**

This prospective cross-sectional study surveyed 247 advanced stage patients (*n* = 122 NET*/n* = 125 CRC) at the National Center for Tumor Diseases, in Germany about cancer-related out-of-pocket costs, income loss, distress, and quality of life. Multiple linear regression analysis was performed to demonstrate the effects of economic deterioration on patients’ quality of life and distress.

**Results:**

81% (*n* = 199) of the patients reported out-of-pocket costs, and 37% (*n* = 92) income loss as a consequence of their disease. While monthly out-of-pocket costs did not exceed 200€ in 77% of affected patients, 24% of those with income losses reported losing more than 1.200€ per month. High financial loss relative to income was significantly associated with patients’ reporting a worse quality of life (*p* < .05) and more distress (*p* < .05).

**Conclusions:**

Financial toxicity in third-party payer health care systems like Germany is caused rather by income loss than by co-payments. Distress and reduced quality of life due to financial problems seem to amplify the burden that already results from a cancer diagnosis and treatment. If confirmed at a broader scale, there is a need for targeted support measures at the individual and system level.

## Background

Research about the impact of cancer on the financial situation of patients has started with a focus on the US healthcare system [1–5]. Studies report that financial hardship is associated with negative physical and psychological effects and can even contribute to an increased mortality rate [6]. As a result, the term ‘financial toxicity’ has been coined, covering both - the objective financial burden from direct and indirect treatment costs and their financial consequences as well as the subjectively perceived distress arising from these costs [7]. A standardized taxonomy and definition of the concept of cancer-related financial impact would contribute to the understanding and comparability of studies on this subject. However, this is still lacking. Based on the current literature, it seems reasonable to use three domains, material, psychosocial and behavioral responses, to measure a patient’s subjective financial distress [8].

So far, little is known about the financial impact of cancer concerning individual patients in third-party payer health care systems like Germany [9]. A recent systematic literature review on studies that measure financial toxicity after cancer diagnosis showed that most studies originate in the US, and only a few come from Europe with none from Germany [8]. Presumably, because disease-related costs in systems with uniform health-care coverage and capped co-payments are not expected to be as relevant as in the US.

Research suggests that financial concerns may affect patient-reported outcomes (PROs) [10]. Studies from the US report financial difficulties to be the most frequent source of distress for cancer patients [11]. They have also been associated with worse quality of life (QoL) [12], worse compliance [13], and lower patient satisfaction [14].

However, first data indicate that financial burden also plays a role in the European health care context: In 2016, an Italian study first showed the association between financial difficulties and relevant cancer patients’ outcomes like QoL and survival [15]. An explorative investigation at the National Center of Tumor Diseases (NCT) by the social counseling service indicated that financial burden is a relevant issue for German cancer patients [16]. A recent study on out-of-pocket costs showed that many cancer patients in Germany face additional disease-related costs, which may burden the affected patients [17].

Apart from out-of-pocket costs, other consequences of cancer disease that affect the family income, such as loss of salary, are conceivable. Therefore, the objective of this study is to characterize the prevalence and intensity of out-of-pocket expenses and income loss and to evaluate their impact on QoL and distress in patients with advanced cancer at a German Comprehensive Cancer Center.

## Methods

### Study design and participants

This is a prospective, cross-sectional survey study that included all consenting German-speaking adult (> 18 years) stage IV patients with either neuroendocrine tumors (NET) or colorectal cancer (CRC) under treatment at the NCT, Heidelberg between 11/2016 and 3/2017. These two groups of cancer patients were selected because, in general, the NET-patient group has a longer disease trajectory compared to patients with metastatic colorectal cancer and might also be younger. Furthermore, NET and CRC are considered the two most prevalent gastrointestinal malignancies [18, 19].

### Survey instrument

In the absence of a German-speaking survey instrument on financial consequences and its association with self-reported outcomes, we designed a questionnaire and pretested it. It was informed by a systematic literature review of our group [8] and included three broad dimensions of individual financial burden: (1) material aspects, (2) psychological effects, and (3) behavioral changes. Additionally, clinical and disease-related parameters were extracted from patients’ records. Overall, the survey instrument contains 23 questions on cancer-related out-of-pocket costs, monthly household income and income loss, distress (NCCN Distress Thermometer [20]), QoL (Q30 of the EORTC QLQ-C30 [21]) and behavioral consequences, as well as demographic data to capture both, direct and indirect cancer-related expenses and the patient’s individual reaction to excess-spending (see Additional file 1). We did not use Q28 of the EORTC QLQ-C30 to measure “financial difficulties”, because it is a single-item question that does not allow distinguishing between financial difficulties generated by functional loss or the medical treatment itself.

We conducted a conventional pretest with 12 cancer patients to check the practicability of the survey process as a whole, especially the comprehensibility of the questions, the functionality of the entire questionnaire as well as the average duration of the questionnaire completion. Based on the results, individual question formulations were sharpened, and missing answer options were added. According to the pretests, the estimated time to complete the survey was 15 min. The participants were informed of this in the declaration of consent. A research assistant handed out the survey to the respondents and was available on-site to answer questions.

Relative loss of income was calculated by converting the ordinal data on loss of income, monthly expenditure, and net household income into monetary units, using the center of the respective interval as the corresponding realized value. Subsequently, the sum of income loss and monthly expenditure was divided by the household net income to obtain an approximation of the relative loss of income. Therapy intensity is a binary variable indicating whether a patient receives chemotherapy, tyrosine kinase inhibitors (TKI), or mechanistic Target of Rapamycin (mTOR) (=1) or not (=0).

### Statistical analysis

The statistical significance of differences between NET and CRC patients was analyzed by the chi-square test for nominal/categorical and t-test for metric levels of measurement. Risk factors for financial losses were identified based on binary logistic regressions. Multivariate linear regression models were fit to assess the relationship between financial loss and distress/QoL. All significance tests were two-sided using a significance level of *p* < .05. Statistical analyses were performed using R, version 3.5.3, and IBM SPSS Statistics, version 22.

## Results

### Demographic and disease-specific data

Two hundred forty-seven of 311 contacted stage IV cancer patients (*n* = 122 NET*/n* = 125 CRC) were included, resulting in a response rate of 79%. The most common reasons for non-response were: not interested (38%), language/understanding difficulties (28%), too nervous before the doctor’s appointment (9%), too exhausted (6%). Median age of the whole sample was 63 years, and 64% of participants were male (see Table 1 for sample characteristics). Time living with metastatic disease was longer in NET patients than in CRC patients (26 vs. 15 months, differences are significant, *p* = .001), and they showed a better performance status (ECOG differs significantly between the two groups, *p* = .035). More CRC patients were unemployed at the time of the survey compared to NET patients (*n* = 21 vs. *n* = 6), the differences in employment status are significant (*p* = .008). All other characteristics presented in Table 1 showed no statistically significant difference.

**Table 1:** Characteristics of study participants

		All (***n*** = 247)	NET (***n*** = 122)	CRC (***n*** = 125)
Sex	Male	159 (64%)	72 (59%)	87 (70%)
	Female	88 (36%)	50 (41%)	38 (30%)
Age [years]	Median	63.0	63.0	63.0
	Range	20–84	20–84	30–80
Age group	≤39	15 (6%)	8 (7%)	7 (6%)
	40–49	25 (10%)	14 (12%)	11 (9%)
	50–59	51 (21%)	21 (17%)	30 (24%)
	60–69	87 (35%)	42 (34%)	45 (36%)
	≥70	69 (28%)	37 (30%)	32 (26%)
Employment status	Employed	74 (30%)	42 (34%)	32 (26%)
	Not employed	27 (11%)	6 (5%)	21 (17%)
	Retired	146 (59%)	74 (61%)	72 (58%)
Duration of illness [months]	Median	26.0	29.5	22.0
	Range	0–252	0–190	1–252
Time since metastastatic disease [months]	Median Range	20.0 0–190	26.0 0–190	15.0 1–141
ECOG	0	114 (46%)	67 (55%)	47 (38%)
	1	113 (46%)	46 (38%)	67 (54%)
	2	19 (8%)	9 (7%)	10 (8%)
	3	1 (0%)	0 (0%)	1 (1%)
Insurance type	Statutory	182 (74%)	88 (72%)	94 (75%)
	Private	57 (23%)	28 (23%)	29 (23%)
	Other/ not specified	8 (3%)	6 (5%)	2 (2%)
Household net income	< 1200€	30 (12%)	10 (8%)	20 (16%)
	1201–2000€	53 (22%)	26 (21%)	27 (22%)
	2001–3000€	49 (20%)	24 (20%)	25 (20%)
	3001–4000€	41 (17%)	23 (19%)	18 (14%)
	4001–5000€	25 (10%)	13 (11%)	12 (10%)
	> 5000€	32 (13%)	16 (13%)	16 (13%)
	Not specified	17 (7%)	10 (8%)	7 (6%)
Number of children living in household	Median Range	0.0 0–5	0.0 0–3	0.0 0–5

### Out-of-pocket costs and income loss

81% of respondents stated that they need to pay out-of-pocket costs related to their disease with a trend towards a larger proportion in CRC patients (84% vs. 77% in NET, not significant). Cancer-related income loss was reported by 37% of patients (see Table 2), with CRC patients being significantly more often affected than NET patients (45% vs. 30%; *p* = .008).

**Table 2:** Prevalence of out-of-pocket costs and income loss

	All (***n*** = 247)	NET (***n*** = 122)	CRC (***n*** = 125)
Out-of-pocket costs			
Yes	199 (81%)	94 (77%)	105 (84%)
Not specified	10 (4%)	5 (4%)	5 (4%)
Income loss			
Yes	92 (37%)	36 (30%)	56 (45%)
Not specified	5 (2%)	2 (2%)	3 (2%)

**Fig. 1:**
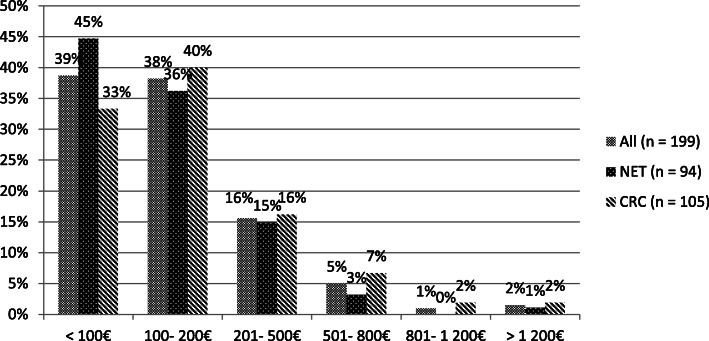
Extent of monthly out-of-pocket costs. Bar chart indicating the amount of monthly out-of-pocket costs (in percent) of all affected patients in comparison to affected neuroendocrine (NET) and colorectal (CRC) cancer patients

**Fig. 2:**
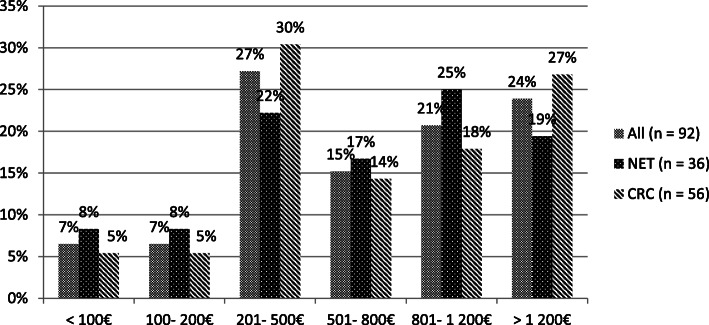
Extent of monthly income loss. Bar chart indicating the amount of monthly income loss (in percent) of all affected patients in comparison to affected neuroendocrine (NET) and colorectal (CRC) cancer patients

77% of the patients with out-of-pocket costs reported amounts not exceeding 200€ monthly, independently of cancer type (see Fig. 1 on average monthly additional expenses incurred due to the tumor disease, i.e., since diagnosis.). The extent of reported average monthly income loss due to the disease was more serious than out-of-pocket costs, as the suffered losses exceeded 1.200€ per month in 24% of affected patients (see Fig. 2). More CRC than NET patients faced high amounts of income loss (27% vs. 19% monthly loss > 1.200€, not significant).

### Consequences of financial losses and associated risk factors

40% of patients reported the need to save money with CRC patients being again more affected than NET patients (45% vs. 34%, differences are not significant). 36% of patients stated to cut back on leisure activities, 14% on nutrition, 10% with regard to living and 9% on non-refundable medication. In all these domains, more CRC patients reported to cut back expenses than those with a NET diagnosis, although not significantly.

Based on binary logistic regression analyses, several risk factors for experiencing financial losses were identified: young age (*p* < .05) and female gender (*p* < .05) were predictive for the presence of out-of-pocket costs. With respect to income loss, young age (*p* < .001) and a CRC disease (*p* < .01) were predictive factors (see Table 3).

**Table 3:** Predictors of out-of-pocket costs

	Binary logistic regression	
	Out-of-pocket costs yes/no (***n*** = 238) regression coefficient B (standard error)	Income loss yes/no (***n*** = 235) regression coefficient B (standard error)
Age (years)	−0.04 (0.02)*	−0.06 (0.01)*
Duration of illness (months)	−0.01 (0.00)	0.01 (0.00)
Primary tumor (CRC = 0, NET = 1)	− 0.64 (0.38)	−0.79 (0.30)*
Sex (m = 0, f = 1)	0.97 (0.46)*	0.02 (0.31)
(Intercept)	5.28 (1.24)*	3.26 (0.80)*

*p < .05

### Subjective financial burden

Multiple regression analysis showed the impact of economic deterioration on patients’ quality of life and distress (see Table 4). In essence, high financial loss relative to income was associated with a lower patient-reported quality of life (*p* < .05) and more distress (*p* < .05). Female gender was also a factor associated with higher distress, whereas a private health insurance scheme was associated with lower distress (*p* < .05).

**Table 4:** Predictors of Quality of life and Distress

Multiple linear regression		
	QoL (***n*** = 216) regression coefficient B (standard error)	Distress (***n*** = 213) regression coefficient B (standard error)
(Intercept)	3.86 (0.71)*	3.70 (1.36)*
Primary tumor (CRC = 0, NET = 1)	0.36 (0.21)	0.11 (0.40)
Sex (m = 0, f = 1)	0.06 (0.19)	1.08 (0.37)*
Age	0.00 (0.01)	−0.00 (0.02)
Number of children living in household	0.08 (0.13)	−0.37 (0.25)
Unemployed (no = 0, yes = 1)	−0.33 (0.31)	− 0.75 (0.60)
Retired (no = 0, yes = 1)	0.09 (0.24)	−0.66 (0.46)
Private health insurance (no = 0, yes = 1)	0.33 (0.20)	−0.80 (0.39)*
Intensity of therapy	−0.32 (0.32)	1.00 (0.61)
Duration of therapy	0.06 (0.08)	−0.15 (0.14)
Duration of illness	−0.00 (0.00)	0.01 (0.00)
Financial loss relative to income	−0.02 (0.00)*	0.02 (0.01)*
R^2^	0.16	0.17
Adj. R^2^	0.11	0.11

*p < .05

## Discussion

The study aimed to explore the prevalence and extent of objective financial burden generated by out-of-pocket expenses or income loss reported by stage IV cancer patients at a German Comprehensive Cancer Center and to evaluate the impact on QoL and distress. So far, the objective financial burden and its subjective consequences have not been systematically investigated for the German healthcare context.

The results of our study show the financial burden of cancer in a large proportion of participating CRC and NET patients: A vast majority of participating CRC and NET patients reported disease-related expenditures. While monthly out-of-pocket costs did not exceed 200€ in most cases, almost half of those with income losses were losing more than 800€ per month. These economic deteriorations showed negative effects on patients’ quality of life and distress. Such findings were unexpected in the public German healthcare system.

Although most medical costs in Germany are covered by a person’s health insurance, patients do have to contribute co-payments for prescription drugs, rehabilitation measures, and hospitalization. Next to these co-payments, many patients face travel expenses to get to the hospital and expenses for non-prescription drugs or housekeeping. Within the German healthcare system, co-payment exemptions apply to particular patient groups. Anyone who reaches a certain limit of co-payments within a calendar year can be exempted from co-payment regulation. The applicable limit of co-payments is 2% of patients’ gross income. In chronically ill patients, this threshold is even lower (1%). Survey results from the US unsurprisingly refer to higher monthly out-of-pocket costs than we found in our investigation, e.g., $806 among 73 advanced head and neck cancer patients in Chicago [22]. Data from the Italian public health systems report 69–244€ costs per month [23]. The particularly high risk of young female patients for out-of-pocket costs in our study might be explained with additional costs for housekeeping or childcare.

Our study shows that in the German social security system, income losses outweigh out-of-pocket costs. A quarter of all affected patients report monthly income losses of 1200€ or more, with CRC patients being significantly more affected than NET patients. As CRC, in general, is a faster-progressing disease than NET, CRC patients are more likely to be limited in their ability to work. A better performance status (ECOG) of enrolled NET patients supports this assumption. As found in other studies, income loss is a major cause of cancer-related financial burden [24]. In Germany, if patients are unable to work due to their illness, their health insurance will pay 70% of their previous income during their sick leave, but no longer than 78 weeks. If there is no return to work, unemployment, or disability pension (approximately 50% of initial income) will follow. Available studies from the US also report that hours worked decline in cancer patients resulting in annual earnings dropping by 40–50% after diagnosis [25, 26]. According to our study, young CRC patients are especially at risk of income loss. Other studies also identified younger age as a factor associated with cancer-related financial problems [27–30]. Cancer treatment may interrupt employment and have a lasting negative impact on earnings and career development. For younger patients who remain fit enough to continue work during treatment, efforts to enable patients to remain in the workforce may help mitigate the financial burden.

40% of patients in our investigation saved money in leisure activities, but also by cutting back on nutrition, living, and medication that is not reimbursed by their health insurance. Comparative studies show that in the US context, a larger amount of patients use lifestyle-altering and even care-altering strategies [31] like taking less than the prescribed amount of medication [13]. While lifestyle changes may not be perceived as physically harmful because they do not require altering a patient’s medical care, they may still be associated with a significant decrease in patients’ quality of life. Future research should address how patients alter spending behaviors and identify potentially harmful cost-coping strategies.

Our study showed the effects of economic deteriorations on PROs with high financial loss relative to income being associated with lower patient-reported quality of life as well as more distress. The result that financial loss is a more significant predictor of quality of life than clinical parameters highlights the potentially powerful impact of financial strain on patients’ overall well-being. This is in line with other studies showing that financial burden negatively impacts patients’ QoL [15, 32–34] and distress [35]. Our analyses also show that the type of insurance (statutory or private) has an influence on the perceived level of distress. Since private health insurance requires an above-average annual income, it can be regarded as a good proxy for a higher socioeconomic status. Exceptions to this rule may be self-employed persons, who can also take out private insurance, but irrespective of an income limit. In general, health insurance is compulsory in Germany. In principle, all employees (or pensioners) whose gross income does not exceed a currently applicable annual income limit are compulsorily insured in the statutory health insurance. For the year 2020, this is 62.550€ or 5.213€ per month. If the annual income is above the compulsory insurance limit, it is possible to take out private health insurance.

Overall, our results suggest that financial burden and its impact on patient-reported outcomes are relevant in a publicly financed health system. We suggest the following next steps for health care, health policy, and research:

First, just as cancer patients receive counseling about nutrition, exercise, and psycho-oncological support, screening for financial risk factors and consecutive counseling should be integrated via the social counseling programs offered to them. The fact that medical out of pocket costs for patients in Germany – and Europe more generally – are relatively low compared to other parts of the world should not lead us to underestimate the importance of their perceived financial burden and leave it solely to the patients to cope with. At the individual level, comprehensive consulting services that contact the patient at an early stage of his or her disease and provide targeted and need-based assistance would be a possible support measure for patients. At the system level, a more flexible sick-pay could be considered (longer than 78 weeks under certain conditions).

Second, there exists no standardized survey instrument for financial burden in the context of the European third-party payer health care systems. Recently published validated instruments from the US like the COST score [36] are not transferable due to relevant differences in the health care systems. Other measurements like Q28 of the EORTC QLQ-C30 do not cover all recently identified domains of individual financial distress [8]. Being the most frequently used disease-specific instrument, the EORTC questionnaire could, however, be used as a rough screening tool to identify cancer indications with a particularly high risk of financial toxicity. An additional instrument reflecting all relevant domains of financial burden should be developed and could then be used within these groups consecutively. A discussion on item domains and taxonomy could be coordinated by the EORTC Quality of Life Group, as they already have experience in developing cancer-specific survey tools, such as the EORTC QLQ-C30, that constitutes an important contribution to the assessment of quality of life of cancer patients.

Third, while register-based data on working capacity, vocational reintegration, income, and need for transfer payments exist in principle, they are inaccessible due to non-cross-linked data pools. A comprehensive analysis and thus, the consideration of population-based data would allow a better differentiation according to gender, age groups, diagnoses, and the different social situations as well as analysis of the temporal development. Therefore, the necessary conditions for nationwide, population, and registry-based research on the socio-economic impact of cancer must be established.

### Limitations

There are several restrictions on the generalizability of this study. First, as it was a monocentric study design with small sample size, the recruited patients represent only a subset of the overall NET and CRC population in Germany. Additionally, the representativeness of the patient sample recruited at the NCT Heidelberg with above-average socio-economic conditions in the commuting area may be limited. However, the fact that considerable financial losses were prevalent even in this selected population suggests that economic challenges are likely to be widespread. Second, although we report a strong response rate from sick patients on a difficult topic, it is possible that our analysis of self-reported variables suffered from nonresponse, recall bias, and social desirability bias. Third, due to the lack of validated instruments that adequately reflect circumstances and reactions to financial constraints within the German social security system, we have used a self-designed instrument. Thus, the results are not directly transferable to other studies. However, they illustrate the need to intensify further input into the development of questionnaires specific to different types of health care and social security systems.

Concerning the compensation of higher out-of-pocket costs or reduced earning capacities, our survey was limited to identify the cost-covering social insurance (e.g. social care) as a proxy for financial liability. Further studies might also try to capture the recourse to financial assistance services (e.g. bank loans) or billing discounts as an additional indicator of financial toxicity. Fourth, our results indicate no direct impact of the number of household members on patients perceived distress resulting from cancer diagnosis and treatment. However, we have not studied other causal relationships, for example, the extent to which other family members are influenced to additional direct and indirect expenditures as a result of cancer. Finally, a cross-sectional study does not allow establishing causal links, so the association we observed between financial loss and QoL/distress needs to be confirmed in other studies with a larger sample size and longitudinal study design.

## Conclusions

Even though cancer treatment costs are primarily covered by the German healthcare system, this study shows that financial loss due to cancer is frequent and is associated with poorer QoL and more distress. Thus, financial loss seems to intensify the burden experienced by patients that already result from a cancer diagnosis. In addition to direct health care costs, which lead to financial burdens, especially in privately organized health care systems, income and earning capacity play an important role in social insurance systems. If validated, these findings provide a rationale for strategies in European health care systems to reduce the financial impact of cancer on patients and their families for example by implementing of early and targeted advice and support measures at the individual level and by introducing more flexible sickness benefit schemes at the system level. For substantiating our findings we therefore advocate for a systematic assessment of financial burden due to cancer disease. To do this, a valid instrument to measure ‘individual financial burden’ in the European context as well as the fostering of population- and registry-based research on the socio-economic impact of cancer are needed.

## Supplementary information

**Additional file 1.** Patient Survey in German language

## Data Availability

The datasets generated and analysed during the current study are not publicly available because individual privacy could be compromised due to potential re-identifiability of individual participants.

## Abbreviations

CRC: Colorectal cancer
mTOR: Mechanistic Target of Rapamycin
NCT: National Center for Tumor Diseases
NET: Neuroendocrine tumor
PROs: Patient-reported outcomes
QoL: Quality of life
TKI: Tyrosine kinase inhibitors

## References

[1] Altice CK, Banegas MP, Tucker-Seeley RD, Yabroff KR. Financial hardships experienced by Cancer survivors: a systematic review. J Natl Cancer Inst. 2017. 10.1093/jnci/djw205. 10.1093/jnci/djw205 PMC6075571 27754926

[2] Azzani M, Roslani AC, Su TT (2015). The perceived cancer-related financial hardship among patients and their families: a systematic review. Support Care Cancer.

[3] Bestvina CM, Zullig LL, Yousuf ZS (2014). The implications of out-of-pocket cost of cancer treatment in the USA: a critical appraisal of the literature. Future Oncol.

[4] Gordon LG, Merollini KMD, Lowe A, Chan RJ (2017). A systematic review of financial toxicity among Cancer survivors: we Can't pay the co-pay. Patient..

[5] Hanratty B, Holland P, Jacoby A, Whitehead M (2007). Financial stress and strain associated with terminal cancer--a review of the evidence. Palliat Med.

[6] Ramsey SD, Bansal A, Fedorenko CR, Blough DK, Overstreet KA, Shankaran V (2016). Financial insolvency as a risk factor for early mortality among patients with Cancer. J Clin Oncol.

[7] Carrera PM, Kantarjian HM, Blinder VS (2018). The financial burden and distress of patients with cancer: understanding and stepping-up action on the financial toxicity of cancer treatment. CA Cancer J Clin.

[8] Witte J, Mehlis K, Surmann B, Lingnau R, Damm O, Greiner W, et al. Methods for measuring financial toxicity after cancer diagnosis and treatment: a systematic review and its implications. Ann Oncol. 2019. 10.1093/annonc/mdz140. 10.1093/annonc/mdz140 PMC6637374 31046080

[9] Huntington SF (2016). Cancer-related financial toxicity: beyond the realm of drug pricing and out-of-pocket costs. Ann Oncol.

[10] Zimmerman FJ, Katon W (2005). Socioeconomic status, depression disparities, and financial strain: what lies behind the income-depression relationship?. Health Econ.

[11] Kendall J, Glaze K, Oakland S, Hansen J, Parry C (2011). What do 1281 distress screeners tell us about cancer patients in a community cancer center?. Psychooncology..

[12] Lathan CS, Cronin A, Tucker-Seeley R, Zafar SY, Ayanian JZ, Schrag D (2016). Association of Financial Strain with Symptom Burden and Quality of life for patients with lung or colorectal Cancer. J Clin Oncol.

[13] Zafar SY, Peppercorn JM, Schrag D, Taylor DH, Goetzinger AM, Zhong X (2013). The financial toxicity of cancer treatment: a pilot study assessing out-of-pocket expenses and the insured cancer patient's experience. Oncologist..

[14] Chino F, Peppercorn J, Taylor DH, Lu Y, Samsa G, Abernethy AP (2014). Self-reported financial burden and satisfaction with care among patients with cancer. Oncologist..

[15] Perrone F, Jommi C, Di Maio M, Gimigliano A, Gridelli C, Pignata S (2016). The association of financial difficulties with clinical outcomes in cancer patients: secondary analysis of 16 academic prospective clinical trials conducted in Italy. Ann Oncol.

[16] Winkler EC, Bikowski K, Schildmann J (2017). Reallocation of resources toward social work and a call for active professionalism. J Clin Oncol.

[17] Büttner M, König HH, Löbner M, Briest S, Konnopka A, Dietz A (2019). Out-of-pocket-payments and the financial burden of 502 cancer patients of working age in Germany: results from a longitudinal study. Support Care Cancer.

[18] Yao JC, Hassan M, Phan A, Dagohoy C, Leary C, Mares JE (2008). One hundred years after "carcinoid": epidemiology of and prognostic factors for neuroendocrine tumors in 35,825 cases in the United States. J Clin Oncol.

[19] Dasari A, Shen C, Halperin D, Zhao B, Zhou S, Xu Y (2017). Trends in the incidence, prevalence, and survival outcomes in patients with neuroendocrine tumors in the United States. JAMA Oncol.

[20] Mehnert A, Müller D, Lehmann C, Koch U (2006). Die deutsche Version des NCCN Distress-Thermometers. Z Psychiatr Psychol Psychother.

[21] Aaronson NK, Ahmedzai S, Bergman B, Bullinger M, Cull A, Duez NJ (1993). The European Organization for Research and Treatment of Cancer QLQ-C30: a quality-of-life instrument for use in international clinical trials in oncology. J Natl Cancer Inst.

[22] de Souza JA, Kung S, O'Connor J, Yap BJ (2017). Determinants of patient-centered financial stress in patients with locally advanced head and neck Cancer. J Oncol Pract..

[23] Baili P, Di Salvo F, de Lorenzo F, Maietta F, Pinto C, Rizzotto V (2016). Out-of-pocket costs for cancer survivors between 5 and 10 years from diagnosis: an Italian population-based study. Support Care Cancer.

[24] Pearce A, Tomalin B, Kaambwa B, Horevoorts N, Duijts S, Mols F (2019). Financial toxicity is more than costs of care: the relationship between employment and financial toxicity in long-term cancer survivors. J Cancer Surviv.

[25] Zajacova A, Dowd JB, Schoeni RF, Wallace RB (2015). Employment and income losses among cancer survivors: estimates from a national longitudinal survey of American families. Cancer..

[26] Khera N, Chang YH, Hashmi S, Slack J, Beebe T, Roy V (2014). Financial burden in recipients of allogeneic hematopoietic cell transplantation. Biol Blood Marrow Transplant.

[27] Barbaret C, Brosse C, Rhondali W, Ruer M, Monsarrat L, Michaud P, et al. Financial distress in patients with advanced cancer. PLoS One. 2017. 10.1371/journal.pone.0176470. 10.1371/journal.pone.0176470 PMC5436643 28545063

[28] Kent EE, Forsythe LP, Yabroff KR, Weaver KE, de Moor JS, Rodriguez JL (2013). Are survivors who report cancer-related financial problems more likely to forgo or delay medical care?. Cancer..

[29] Shankaran V, Jolly S, Blough D, Ramsey SD (2012). Risk factors for financial hardship in patients receiving adjuvant chemotherapy for colon cancer: a population-based exploratory analysis. J Clin Oncol.

[30] Yabroff KR, Dowling EC, Guy GP, Banegas MP, Davidoff A, Han X (2016). Financial hardship associated with Cancer in the United States: findings from a population-based sample of adult Cancer survivors. J Clin Oncol.

[31] Nipp RD, Zullig LL, Samsa G, Peppercorn JM, Schrag D, Taylor DH (2016). Identifying cancer patients who alter care or lifestyle due to treatment-related financial distress. Psychooncology..

[32] Fenn KM, Evans SB, McCorkle R, DiGiovanna MP, Pusztai L, Sanft T (2014). Impact of financial burden of cancer on survivors' quality of life. J Oncol Pract.

[33] Gupta D, Lis CG, Grutsch JF (2007). Perceived cancer-related financial difficulty: implications for patient satisfaction with quality of life in advanced cancer. Support Care Cancer.

[34] Kimman M, Jan S, Monaghan H, Woodward M (2015). The relationship between economic characteristics and health-related quality of life in newly diagnosed cancer patients in Southeast Asia: results from an observational study. Qual Life Res.

[35] Sharp L, Carsin AE, Timmons A (2013). Associations between cancer-related financial stress and strain and psychological well-being among individuals living with cancer. Psychooncology..

[36] de Souza JA, Yap BJ, Hlubocky FJ, Wroblewski K, Ratain MJ, Cella D (2014). The development of a financial toxicity patient-reported outcome in cancer: the COST measure. Cancer..

