# Supplementary material for: The patient-level effect of the cost of Cancer care – financial burden in German Cancer patients
Source: BMC Cancer. 2020 Jun 5;20:529. doi: 10.1186/s12885-020-07028-4 (PMC7275553; doi:10.1186/s12885-020-07028-4)
Supplement: Supplementary file 1 — Additional file 1. Patient Survey in German language [file 12885_2020_7028_MOESM1_ESM.pdf]

Patientenfragebogen zum Forschungsprojekt am NCT

„Wirtschaftliche Auswirkungen einer Krebserkrankung -
Untersuchung am Beispiel von Patienten mit neuroendokrinen Tumoren oder Kolonkarzinomen“

A) Soziodemographische Daten

1. Geschlecht

- ☐ männlich
- ☐ weiblich

2. Wie alt sind Sie?

______________ Jahre

3. Wie viele Personen leben derzeit in Ihrem Haushalt?

- ☐ Ich lebe alleine
- ☐ Ich lebe zusammen mit meinem Lebenspartner/meiner Lebenspartnerin
- ☐ Ich lebe zusammen mit meinem Ehepartner/meiner Ehepartnerin
- ☐ Ich bin alleinerziehend

Im Haushalt leben minderjährige Kinder: ____________ (Anzahl)

4. Ihr höchster Schulabschluss

- ☐ kein Abschluss
- ☐ Hauptschulabschluss/Volksschule
- ☐ Fachoberschulreife/Realschulabschluss
- ☐ Fachhochschulreife/Fachabitur
- ☐ Hochschulreife/Abitur
- ☐ Sonstiges:______________________

5. Leiden Sie an weiteren Erkrankungen neben Ihrer Krebserkrankung?

- ☐ ja, und zwar:____

  ____
- ☐ nein

B) Berufliche Tätigkeit und Einkommen

6. Wie ist ihr derzeitiger Erwerbsstatus?

- ☐ angestellt beschäftigt
- ☐ selbständig oder freiberuflich tätig
- ☐ Beamter/Beamtin
- ☐ teilzeiterwerbstätig
- ☐ nicht erwerbstätig
- ☐ berentet

a) Wenn Sie derzeit erwerbstätig sind – handelt es sich dabei um ein befris-tetes oder unbefristetes Arbeitsverhältnis?

- ☐ Ich habe einen befristeten Arbeitsvertrag
- ☐ Ich habe einen unbefristeten Arbeitsvertrag

b) Wenn Sie nicht erwerbstätig sind, was ist der Grund hierfür?

- ☐ Ich bin arbeitssuchend
- ☐ Ich kann nicht erwerbstätig sein aufgrund von Krankheit
- ☐ Ich möchte nicht erwerbstätig sein
- ☐ Sonstiges: ____

7. Seit wie vielen Monaten sind Sie krankgeschrieben?

- ☐ ______________ Monate
- ☐ Ich bin nicht krankgeschrieben

8. Haben Sie ein eigenes Einkommen?

- ☐ ja
- ☐ nein

a) Wenn ja, wie hoch ist Ihr eigenes monatliches Nettoeinkommen, also die Summe, die nach Abzug der Steuern und Sozialversicherungsbeiträge übrigbleibt?

- ☐ unter 707 Euro
- ☐ 707 - 987 Euro
- ☐ 988 - 1.151 Euro
- ☐ 1.152 - 1.644 Euro
- ☐ 1.645 - 2.467 Euro
- ☐ über 2.467 Euro

9. Sind von Ihrem Einkommen weitere Personen abhängig?

- ☐ ja, und zwar:__________(Anzahl) weitere Personen
- ☐ nein, nur ich

10. Wie hoch ist Ihr gesamtes verfügbares monatliches Netto-Familieneinkommen?

- ☐ unter 1.300 Euro
- ☐ 1.301 - 1.700 Euro
- ☐ 1.701 - 2.600 Euro
- ☐ 2.601 - 3.600 Euro
- ☐ 3.601 - 5.000 Euro
- ☐ über 5.000 Euro

11. Wie sind Sie krankenversichert?

- ☐ Privat
- ☐ Gesetzlich
- ☐ Beitragsfrei mitversichert
- ☐ anders, und zwar:____

C) Einkommen und Ausgaben seit Erkrankung

12. Sind Ihnen durch Ihre Tumorerkrankung Einkommenseinbußen entstanden?

- ☐ ja
- ☐ nein

a) Wenn ja, in welcher Höhe monatlich?

- ☐ unter 100 Euro
- ☐ 100 - 200 Euro
- ☐ 201 - 500 Euro
- ☐ 501 - 800 Euro
- ☐ 801 - 1.200 Euro
- ☐ über 1.200 Euro

b) Wenn ja, haben Sie die Möglichkeit, diese Einbußen auszugleichen?
(Mehrfachnennungen möglich)

- ☐ ja, über Kredit
- ☐ ja, über Ersparnisse oder Vermögen
- ☐ ja, über Sonstiges: ______________________________________
- ☐ nein

13. Haben Sie infolge Ihrer Erkrankung höhere Ausgaben? (z. B. Taxikosten, Zuzahlung für Arzneimittel, Behandlungen)

- ☐ ja
- ☐ nein

a) Wenn ja, in welcher Höhe monatlich?

- ☐ unter 100 Euro
- ☐ 100 - 200 Euro
- ☐ 201 - 500 Euro
- ☐ 501 - 800 Euro
- ☐ 801 - 1.200 Euro
- ☐ über 1.200 Euro

b) Wenn ja, wodurch fallen Ihnen höhere Ausgaben an?
(Mehrfachnennungen möglich)

- ☐ Zuzahlungen
- ☐ Fahrtkosten
- ☐ Inanspruchnahme von Haushaltshilfen
- ☐ Behandlungen und Medikamente, die nicht von der Krankenkasse erstattet werden
- ☐ Sonstiges: ____

14. Hat Ihre Erkrankung zur Verschlechterung Ihrer Lebensverhältnisse insgesamt geführt?

- ☐ ja
- ☐ nein

a) Wenn ja, wo konkret müssen Sie sparen? *(Mehrfachnennungen möglich)*

- ☐ in meinem Freizeitverhalten (z. B. Kinobesuche, Urlaub)
- ☐ bei meiner Ernährung/Lebensmittel
- ☐ beim Wohnen/im Haushalt (z. B. Miete, Strom, Heizung)
- ☐ bei medizinischen Behandlungen/Zusatzleistungen
- ☐ Sonstiges: ____

15. Haben Sie den Behandlungsplan verändert, um weiterhin Ihrem Beruf nachgehen zu können?

- ☐ ja
- ☐ nein

16. Konnten Sie Ihr berufliches Umfeld ändern, um den Behandlungsplan zu erfüllen? *(Mehrfachnennungen möglich)*

- ☐ ja, durch Jobwechsel
- ☐ ja, durch Reduzierung der Arbeitszeit (z. B. Teilzeit)
- ☐ ja, durch Arbeit von zuhause/Home-Office
- ☐ ja, durch Sonstiges: ____
- ☐ nein

17. Welche der folgenden Leistungen erhalten Sie zurzeit?

- ☐ Lohn/Gehalt
- ☐ Rente
- ☐ Krankengeld
- ☐ Erwerbsminderungsrente
- ☐ ALG I
- ☐ ALG II/Hartz IV
- ☐ Grundsicherung
- ☐ Sozialhilfe
- ☐ Leistungen aus einer Berufsunfähigkeitsversicherung
- ☐ Sonstige, und zwar: ____

18. Haben/Hatten Sie Schwierigkeiten oben genannte Leistungen zu erhalten?

- ☐ ja, weil:____
- ☐ nein

19. Haben Sie einen Rehabilitationsantrag/Rentenantrag gestellt?

- ☐ ja
- ☐ nein

a) Wenn ja, wie kam es dazu?

- ☐ Meine Krankenkasse hat mich dazu aufgefordert
- ☐ Mein Arbeitgeber hat mich dazu aufgefordert
- ☐ Ich habe den Antrag von mir aus gestellt

D) Gesundheit

20. Bitte kreuzen Sie bei den folgenden Fragen die Zahl zwischen 1 und 7 an, die am besten auf Sie zutrifft.

Wie würden Sie insgesamt Ihren körperlichen Zustand während der letzten Woche einschätzen?

1 2 3 4 5 6 7

Sehr schlecht ausgezeichnet

Wie würden Sie insgesamt Ihre Lebensqualität während der letzten Woche einschätzen?

1 2 3 4 5 6 7

Sehr schlecht ausgezeichnet

21. Bitte kreuzen Sie unter jeder Überschrift DAS Kästchen an, das Ihre Gesundheit HEUTE am besten beschreibt.

BEWEGLICHKEIT / MOBILITÄT

- ☐ Ich habe keine Probleme herumzugehen
- ☐ Ich habe leichte Probleme herumzugehen
- ☐ Ich habe mäßige Probleme herumzugehen
- ☐ Ich habe große Probleme herumzugehen
- ☐ Ich bin nicht in der Lage herumzugehen

FÜR SICH SELBST SORGEN

- ☐ Ich habe keine Probleme, mich selbst zu waschen oder anzuziehen
- ☐ Ich habe leichte Probleme, mich selbst zu waschen oder anzuziehen
- ☐ Ich habe mäßige Probleme, mich selbst zu waschen oder anzuziehen
- ☐ Ich habe große Probleme, mich selbst zu waschen oder anzuziehen
- ☐ Ich bin nicht in der Lage, mich selbst zu waschen oder anzuziehen

ALLTÄGLICHE TÄTIGKEITEN *(z. B. Arbeit, Studium, Hausarbeit, Familien- oder Freizeitaktivitäten)*

- ☐ Ich habe keine Probleme, meinen alltäglichen Tätigkeiten nachzugehen
- ☐ Ich habe leichte Probleme, meinen alltäglichen Tätigkeiten nachzugehen
- ☐ Ich habe mäßige Probleme, meinen alltäglichen Tätigkeiten nachzugehen
- ☐ Ich habe große Probleme, meinen alltäglichen Tätigkeiten nachzugehen
- ☐ Ich bin nicht in der Lage, meinen alltäglichen Tätigkeiten nachzugehen

SCHMERZEN / KÖRPERLICHE BESCHWERDEN

- ☐ Ich habe keine Schmerzen oder Beschwerden
- ☐ Ich habe leichte Schmerzen oder Beschwerden
- ☐ Ich habe mäßige Schmerzen oder Beschwerden
- ☐ Ich habe starke Schmerzen oder Beschwerden
- ☐ Ich habe extreme Schmerzen oder Beschwerden

ANGST / NIEDERGESCHLAGENHEIT

- ☐ Ich bin nicht ängstlich oder deprimiert
- ☐ Ich bin ein wenig ängstlich oder deprimiert
- ☐ Ich bin mäßig ängstlich oder deprimiert
- ☐ Ich bin sehr ängstlich oder deprimiert
- ☐ Ich bin extrem ängstlich oder deprimiert

Wir wollen herausfinden, wie gut oder schlecht
Ihre Gesundheit **HEUTE** ist.

Diese Skala ist mit Zahlen von 0 bis 100 versehen.
100 ist die beste Gesundheit, die Sie sich vorstellen können.
0 (Null) ist die schlechteste Gesundheit, die Sie sich vorstellen können.

Bitte kreuzen Sie den Punkt auf der Skala an, der Ihre Gesundheit HEUTE am besten beschreibt.

Jetzt tragen Sie bitte die Zahl, die Sie auf der Skala angekreuzt haben, in das Kästchen unten ein:

IHRE GESUNDHEIT HEUTE = [ ]

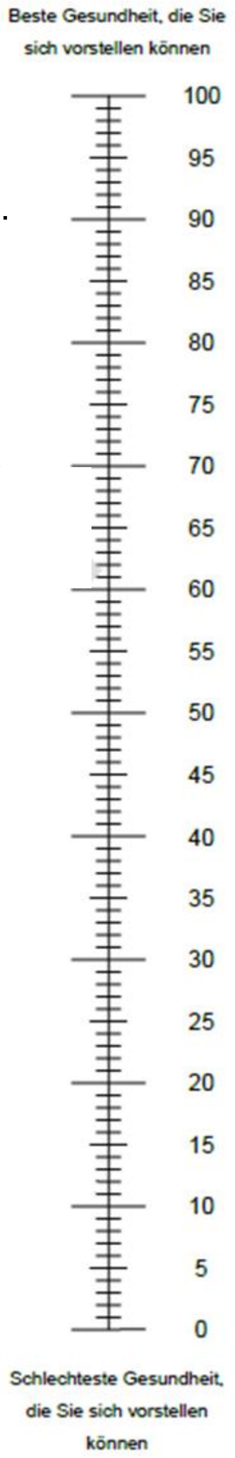

E) Belastung

22. Wie schätzen Sie die Belastung durch Ihre Erkrankung ein?
Bitte umkreisen Sie die Zahl des "Thermometers", die am besten beschreibt, wie belastet Sie sich durch Ihre Erkrankung in der letzten Woche einschließlich heute gefühlt haben.

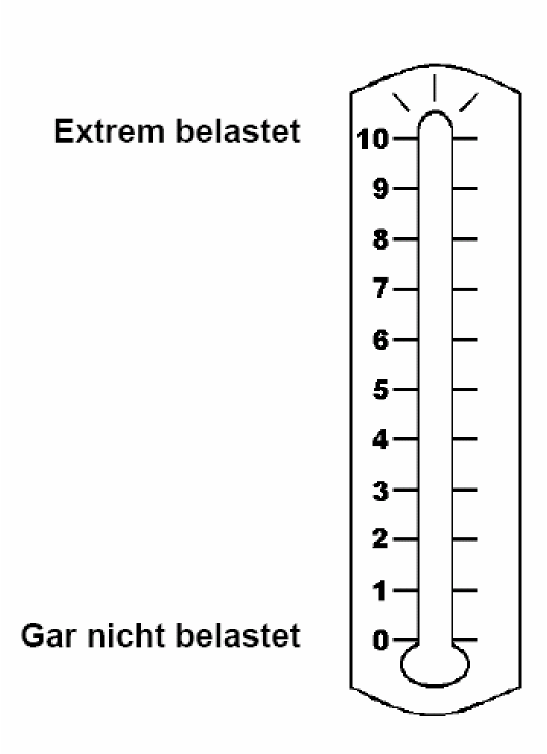

a) **Bitte geben Sie an, ob Sie in einem der nachfolgenden Bereiche in der letzten Woche einschließlich heute Probleme hatten.** Kreuzen Sie bitte für jeden Bereich ja oder nein an.

Praktische Probleme
ja nein
☐ ☐ Wohnsituation
☐ ☐ Versicherung
☐ ☐ Arbeit / Schule
☐ ☐ Beförderung (Transport)
☐ ☐ Kinderbetreuung

Emotionale Probleme
ja nein
☐ ☐ Sorgen
☐ ☐ Ängste
☐ ☐ Traurigkeit
☐ ☐ Depression
☐ ☐ Nervosität

Familiäre Probleme
ja nein
☐ ☐ Im Umgang mit dem Partner
☐ ☐ Im Umgang mit den Kindern

Spirituelle / religiöse Belange
ja nein
☐ ☐ In Bezug auf Gott
☐ ☐ Verlust des Glaubens

Körperliche Probleme
ja nein
- ☐ ☐ Schmerzen
- ☐ ☐ Übelkeit
- ☐ ☐ Erschöpfung
- ☐ ☐ Schlaf
- ☐ ☐ Bewegung / Mobilität
- ☐ ☐ Waschen, Ankleiden
- ☐ ☐ Äußeres Erscheinungsbild
- ☐ ☐ Atmung
- ☐ ☐ Entzündungen im Mundbereich
- ☐ ☐ Essen / Ernährung

Körperliche Probleme
ja nein
- ☐ ☐ Verdauungsstörungen
- ☐ ☐ Verstopfung
- ☐ ☐ Durchfall
- ☐ ☐ Wasserlassen
- ☐ ☐ Fieber
- ☐ ☐ Trockene / juckende Haut
- ☐ ☐ Trockene / verstopfte Nase
- ☐ ☐ Kribbeln in Händen / Füßen
- ☐ ☐ sich aufgedunsen fühlen
- ☐ ☐ Sexuelle Probleme

23. Haben Sie zu diesem Themenbereich Beratungsbedarf?

☐ ja

➔ Wenden Sie sich bitte an die NCT Beratungsangebote.
Kontakt über NCT Sozialdienst, Telefon: +49 6221 564477
oder per E-Mail: sozialarbeit@nct-heidelberg.de

☐ nein

Herzlichen Dank für Ihre Teilnahme!
